# Identifying potential maternal genes of *Bombyx mori* using digital gene expression profiling

**DOI:** 10.1371/journal.pone.0192745

**Published:** 2018-02-20

**Authors:** Meirong Zhang, Sheng Qin, Pingzhen Xu, Guozheng Zhang

**Affiliations:** 1 School of Biotechnology, Jiangsu University of Science and Technology, Zhenjiang Jiangsu, China; 2 Sericulture Research Institute, Chinese Academy of Agricultural Sciences, Zhenjiang Jiangsu, China; University of Kentucky, UNITED STATES

## Abstract

Maternal genes present in mature oocytes play a crucial role in the early development of silkworm. Although maternal genes have been widely studied in many other species, there has been limited research in *Bombyx mori*. High-throughput next generation sequencing provides a practical method for gene discovery on a genome-wide level. Herein, a transcriptome study was used to identify maternal-related genes from silkworm eggs. Unfertilized eggs from five different stages of early development were used to detect the changing situation of gene expression. The expressed genes showed different patterns over time. Seventy-six maternal genes were annotated according to homology analysis with *Drosophila melanogaster*. More than half of the differentially expressed maternal genes fell into four expression patterns, while the expression patterns showed a downward trend over time. The functional annotation of these material genes was mainly related to transcription factor activity, growth factor activity, nucleic acid binding, RNA binding, ATP binding, and ion binding. Additionally, twenty-two gene clusters including maternal genes were identified from 18 scaffolds. Altogether, we plotted a profile for the maternal genes of *Bombyx mori* using a digital gene expression profiling method. This will provide the basis for maternal-specific signature research and improve the understanding of the early development of silkworm.

## Introduction

Maternal genes whose RNA or protein products present in oocytes, fertilized egg or early stage of embryo play an important role in the proper developmental stages [1, 2]. In the early stages of development, the transition of material to the zygote is accomplished by two processes: the degradation of maternal products and the initiation of the transcription of the zygotic genome [3–5]. Maternal genes have been well studied in many species, such as humans, mouse, zebrafish and so on [3, 5–9]. In insects, maternal genes and their functions were well known across various *Drosophila* species [10–14].

*Bombyx mori* (*B*. *mori*) is an important economic insect in agriculture. The development of *B*. *mori* is significantly different from *Drosophila*. RFLP linkage analysis showed that maternal genes might locate at all twenty-eight chromosomes of *B*. *mori* [15]. However, only a few maternal genes have been researched sufficiently. For example, *BmCad* was a homologue of *Drosophila Cad* which revealed a very similar but distinct genetic hierarchy compared to *Drosophila* [16]. *BmHNF-4* transcribed two isoforms as components of follicular cell differentiation [17]. The polypeptides encoded by the *BmHNF-4* gene were required during both early and more advanced stages of embryogenesis. *BmVLG*, whose mRNA or protein products were deposited in the oocyte cytoplasm, was expressed in both unfertilized eggs and embryonic stages of fertilized eggs [18]. Although these studies demonstrated the functions of maternal genes during the development of the zygote, it is still limited to explain the molecular mechanisms of early oogenesis and the embryogenesis of the silkworm.

**Fig 1:**
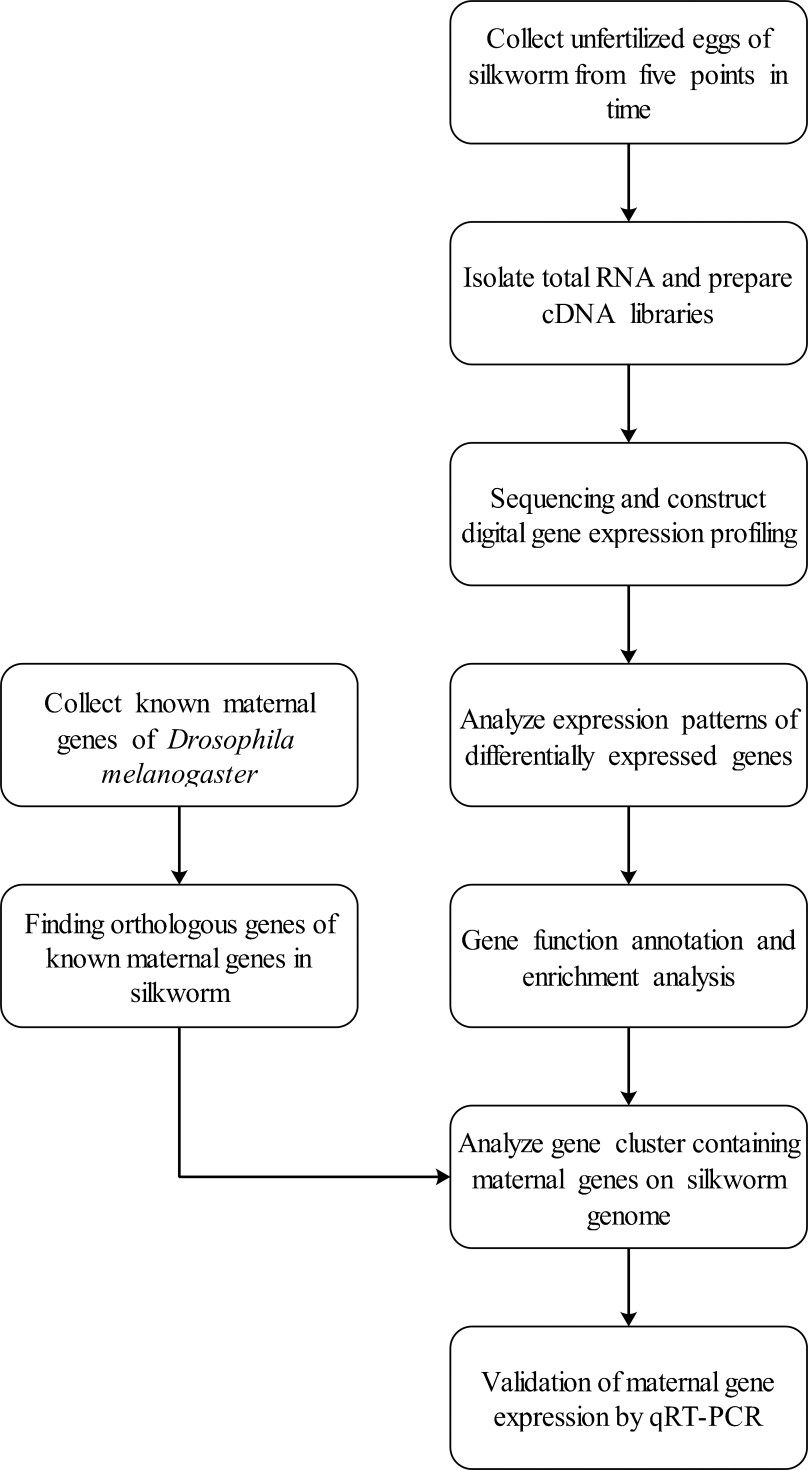
Workflow. Experimental design and schematic diagram of the workflow to identify maternal genes of *Bombyx mori*.

In this study, we identified potential maternal genes in silkworm. Also, the expression profiles of maternal genes were determined 24 h after spawning. The whole workflow is as follows (**Fig 1)**:

First, unfertilized eggs were collected from five points-in-time after spawning. Second, total RNA was isolated and cDNA libraries were constructed using the sample of each time. After that, analyses of digital gene expression profiling were performed on all cDNA libraries and 12,264 genes were annotated. The orthologous genes with the known maternal genes of *Drosophila melanogaster* were analyzed as potential maternal genes of *B*. *mori*. The expression patterns of these orthologous maternal genes were analyzed, and the molecular functions of differentially expressed genes was annotated. This study will provide a new perspective for understanding the early development of silkworm.

## Materials and methods

### Sample preparation

The *B*. *mori* strain Dazao was obtained from the Sericulture Research Institute of China Academy of Agricultural Sciences. The larvae were reared under standard conditions until spawning (25°C and 70% humidity). Unfertilized and fertilized embryos were collected at specific points-in-time (0 h, 6 h, 12 h, 18 h, 24 h). The fertilized embryos of five time points were only used to real-time quantitative PCR analysis. 0 h is defined as the 15^th^ minutes after most female moths spawn. The conditions during embryo collection were 25°C and 70% humidity with 12-h light/12-h dark photoperiod. All samples were immediately stored in liquid nitrogen.

### RNA extraction

Total RNA was extracted from embryos at various times using the total RNA isolation kit (Qiagen, USA) according to the manufacturer’s instructions. The mRNA was purified by Oligo(dT) magnetic beads (Invitrogen, USA), and then used as templates for cDNA synthesis.

### Digital gene expression profile

The 5' end of tags which recognized and cut off the CATG sites on cDNAs were generated by endonuclease Nla III. The fragments apart from the 3' cDNA fragments connected to Oligo(dT) beads were washed away and the Illumina adaptor 1 was ligated to the sticky 5' end of the digested bead-bound cDNA fragments. The junction of Illumina adaptor 1 and CATG site was the recognition site of MmeI, which was a type of endonuclease with separated recognition sites and digestion sites. It cut 17bp downstream of the CATG site to produce tags with adaptor 1. After removing 3' fragments with magnetic beads precipitation, Illumina adaptor 2 was ligated to the 3' ends of tags to acquire tags with different adaptors of both ends to form a tag library. The raw tags were sequenced by the Illumina HiSeq^TM^ 2000. Clean tags were generated by removing the 3' adaptor sequence, low quality tags, tags with a copy number of 1 and tags which were too long or too short, leaving only tags which were 21 bp long.

### Annotation of expressed genes

A virtual library containing all of the possible CATG+17 bases sequences of the reference gene sequences were constructed based on the silkworm database (ftp://silkdb.org) for gene annotation. All clean tags were mapped to the reference sequences and only 1bp mismatches were considered. Clean tags mapped to reference sequences from multiple genes were filtered. The remaining clean tags were designated unambiguous clean tags. Unambiguous clean tags were used to identify genes and assess the expression level of genes. A gene mapped by at least one count tag is defined as an expressed gene.

### Identification of differentially expressed genes (DEGs)

The DEGs in the samples were identified based on digital gene expression profiling following the method of Audic S *et al*. [19]. Multiple-test correction was performed to control the false discovery rate (FDR) [20]. Genes with FDR≤0.01 and |log_2_ Ratio|≥1 were defined as the DEGs. The DEGs were obtained following two strategies: (I) The sample from four time points, except 0 h, was compared with the sample from 0h. (II) Each sample was compared with the sample of the prior point in time.

### Orthologous analysis

Maternal genes of *Drosophila melanogaster* were obtained from NCBI based on the retrieve conditions "(maternal) AND 7227[Taxonomy ID]". Orthologous genes in *B*. *mori* were collected from OrthoDB v9 [21].

### Genes expression patterns among different time points

Based on strategy I, a gene with a log2 Ratio of TPM for all time points would be selected when its expression levels were significantly different in any comparison. The expression patterns were clustered using hierarchical cluster analysis. The Pearson's correlation of log2 Ratio among the genes was used as the distance for clustering.

### GO annotation and enrichment analyses

All 14,623 genes obtained from silkworm database were annotated for protein function using InterProScan 5.19.58 (http://www.ebi.ac.uk/interpro/) with the "-goterms" parameter [22]. The GO enrichment was evaluated using the Fisher’s exact test function implemented in Blast2GO (*P*≤0.01).

### Real-time quantitative PCR analysis

In order to analyze the orthologous maternal differentially expressed genes (OMDEGs) identified by our transcriptome sequencing analysis between unfertilized and fertilized embryos, reverse transcription quantitative PCR (RT-qPCR) was adopted. Total RNA from each sample was used to synthesize the first strand cDNA using the PrimeScript Reverse Transcriptase kit (TaKaRa, China) according to the instructions of the manufacturer. RT-qPCR was carried out in an ABI PRISM® 7300 Sequence Detection System (Applied Biosystems) using SYBR Green Supermix (TaKaRa, China) according the instructions of the manufacturer. The thermal cycling conditions profile consisted of an initial denaturation at 94°C for 10 min, followed by 40 cycles of 94°C for 15 s, and 60°C for 31 s, and then an extension. The expression levels were calculated using the 2^-ΔΔCT^ method. *Bmactin3* was used as a reference gene.

## Results

### Summary of digital gene expression (DGE) profiles

Five time-series samples were prepared which were collected at 0 h, 6 h, 12 h, 18 h and 24 h after unfertilized embryo spawning. DGE tag libraries were constructed and sequenced using the Illumina HiSeq^TM^ 2000. In total, 5.7–6.0 million raw tags were generated from each library, 96.97–98.07% of which were filtered as clean tags after quality control. Among the clean tags of each sample, a total of 0.08–0.11 million tags were distinct. The details of tag sequencing are listed in **Table 1**. In each sample, 7–9% of distinct clean tags had more than 100 copies, 35–38% of them had 6 to 100 copies and 54–57% of had 2 to 5 copies. This indicates that most of them were expressed at a low level and a small part of them were expressed at a very high level. The raw data have been submitted to the GEO database with the accession number of GSE109815.

**Table 1:** Summary of tags and mapped genes in the DEG libraries.

Summary		0h	6h	12h	18h	24h
**Raw Data**	Total	6033953	5896636	5782715	5865535	5785000
	Distinct Tag	282267	280867	277144	258012	191942
**Clean Tag**	Total number	5854282	5717831	5607394	5704932	5673650
	Distinct Tag number	105259	104536	104115	99597	82971
**All Tag-Mapping to Gene**	Total number	769935	791770	814159	1034126	1270324
	Total % of clean tag	13.15%	13.85%	14.52%	18.13%	22.39%
	Distinct Tag number	18456	19755	18374	18812	16892
	Distinct Tag % of clean tag	17.53%	18.90%	17.65%	18.89%	20.36%
**Unambiguous Tag-Mapping to Gene**	Total number	755083	778145	805111	1020601	1251238
	Total % of clean tag	12.90%	13.61%	14.36%	17.89%	22.05%
	Distinct Tag number	18217	19505	18139	18549	16626
	Distinct Tag % of clean tag	17.31%	18.66%	17.42%	18.62%	20.04%
**All Tag-mapped Genes**	number	6374	6457	6344	6732	6133
	% of ref genes	43.59%	44.16%	43.38%	46.04%	41.94%
**Unambiguous Tag-mapped Genes**	number	6183	6286	6193	6516	5912
	% of ref genes	42.28%	42.99%	42.35%	44.56%	40.43%
**Mapping to Genome**	Total number	4187834	4100044	3947447	3827770	3634083
	Total % of clean tag	71.53%	71.71%	70.40%	67.10%	64.05%
	Distinct Tag number	58257	58143	57482	58627	50707
	Distinct Tag % of clean tag	55.35%	55.62%	55.21%	58.86%	61.11%
**Unknown Tag**	Total number	896513	826017	845788	843036	769243
	Total % of clean tag	15.31%	14.45%	15.08%	14.78%	13.56%
	Distinct Tag number	28546	26638	28259	22158	15372
	Distinct Tag % of clean tag	27.12%	25.48%	27.14%	22.25%	18.53%

### Analysis of tag mapping

For tag mapping, a reference tag database containing 54,721 unambiguous reference tags and 12,264 genes with CATG sites was constructed based on the silkworm database (ftp://silkdb.org). In total, 17–20% of distinct tags in five DGE libraries were mapped to the genes and 40–45% of known silkworm genes were expressed in the sampled oocytes according to matched unambiguous tags (**Table 1**). In the remaining district tags, 55–61% were mapped to the silkworm genome; 19–27% of tags distinguished as unknown could not be mapped to the reference tag library.

### Differentially expressed genes among different times

To identify genes with a significant change in expression during different developmental stages, the expression abundances of tag-mapped genes were normalized by counting the number of transcripts per million (TPM) clean tags. Tag abundances and TPM values of expressed genes are shown in **S1 Table**.

The DEGs among five samples were filtered with the threshold of FDR ≤0.01 and the |log_2_ Ratio|≥1. Gene expressions were compared based on two strategies, as described above.

**Fig 2:**
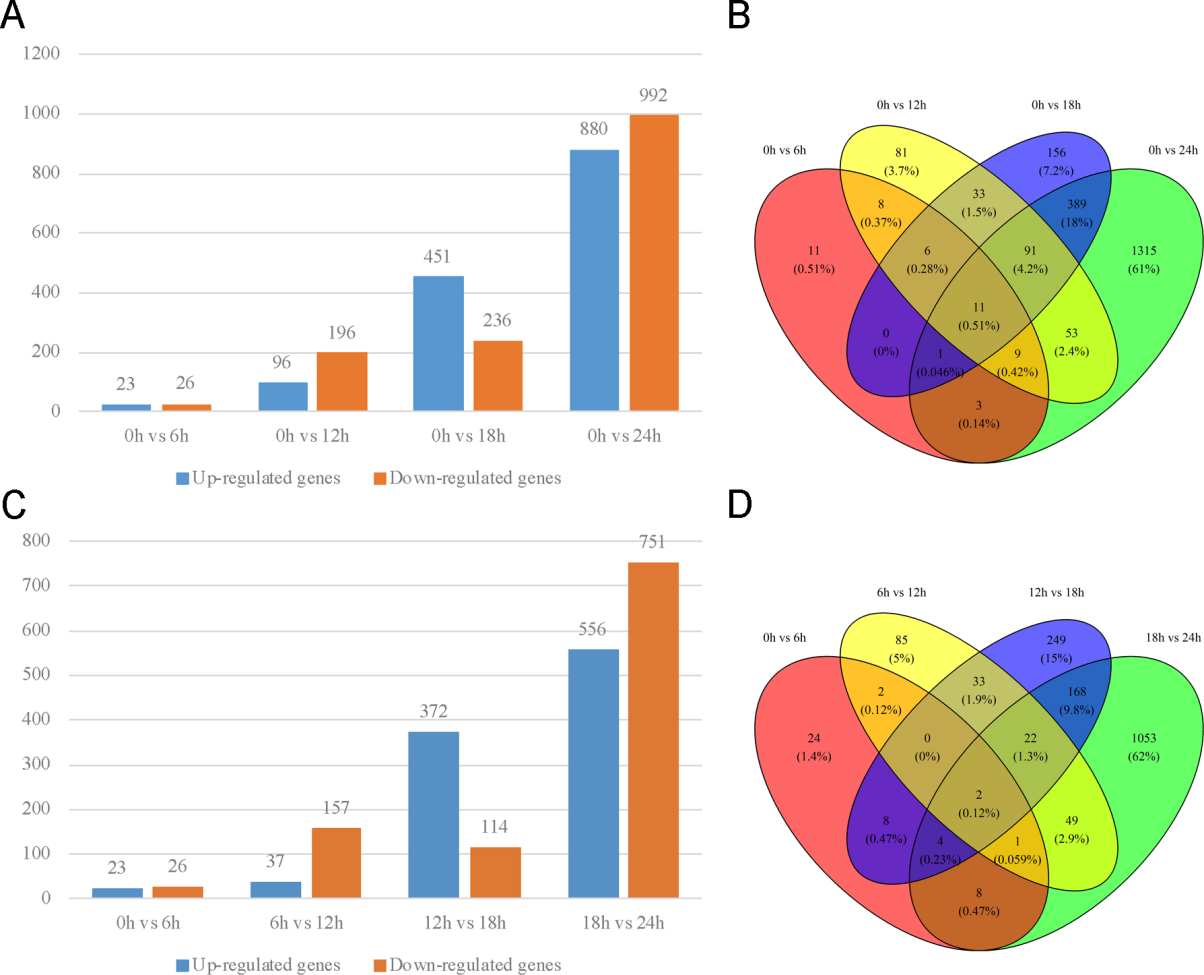
Summary of differentially expressed genes among five points in time. (A) Compared with 0 h, up-regulated and down-regulated genes for the other four times. (B) Venn diagram of differentially expressed genes in Fig 2A. (C) Compared with prior time point, up-regulated and down-regulated genes for the current time point. (D) Venn diagram of differentially expressed genes in Fig 2C. Blue bar indicates up-regulated gene and orange bar indicates down- regulated gene.

Based on strategy I, the results showed that the number of DEGs was increased with time (**Fig 2A**). In total 2,167 genes were identified as DEGs at each time point of 6 h, 12 h, 18 h and 24 h. These DEGs and their log2-fold changes of expression were used for clustering analysis in the next step (**S2 Table**). Eleven common genes were differentially expressed across all four time points (**Fig 2B**).

Based on strategy II, a similar trend was observed, where the number of DEGs increased with time (**Fig 2C**). A total of 372 genes showed significant up-regulation from 12 h to 18 h, but 751 genes showed significant down-regulation from 18 h to 24 h. There were 57 common DEGs between the 6 h and 12 h groups and the 12 h and 18 h groups. In total, 85.96% of them were down-regulated from 6 h to 12 h and 91.23% were up-regulated from 12 h to 18 h (**Fig 2D**). Conversely, there were 196 common DEGs between the 12 h vs. 18 h groups and the 18 h vs. 24 h groups. Overall, 79.08% were up-regulated from 12 h to 18 h, while 51.53% of them maintained high expression levels from 18 h to 24 h. Only two common genes were differentially expressed consistently over the entire period.

### Orthologous maternal genes (OMGs) between *Drosophila melanogaster* and *Bombyx mori*

Ninety-nine maternal genes from *Drosophila melanogaster* were collected from the NCBI database. Fifty-one were found to be consistent with 76 OMGs in the *B*. *mori* genome (**S3 Table**). Sixty OMGs were detected as expressed genes, with twenty-three of them being DEGs according to strategy I. The expression levels of 56.52% (13/23) of orthologous maternal DEGs (OMDEGs) were up-regulated for more than 10 TPM for at least one point-in-time, but this proportion of orthologous maternal non-DEGs was only 16.22% (6/37). This indicates that most non-DEG maternal genes were poorly expressed even almost unexpressed.

### Analysis of time-series gene expression patterns

**Fig 3:**
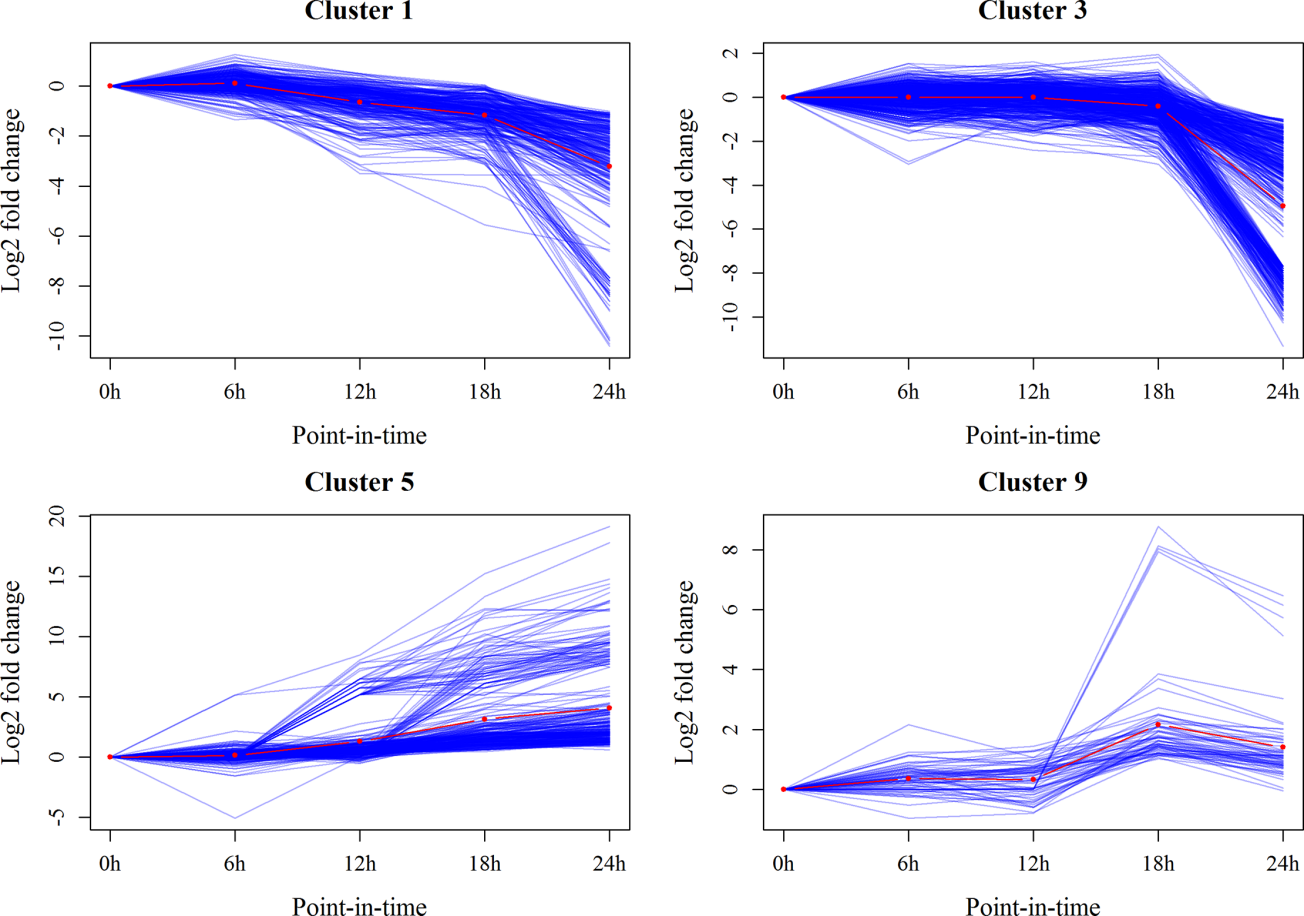
Four expression patterns of differentially expressed genes containing more than two maternal genes. Blue lines indicate the expression pattern of genes in current cluster. Red line indicates the average expression level of genes.

The majority of expressed genes showed time-specific expression patterns and could be clustered into 27 distinct groups (**S1 Fig**). Twenty-three OMDEGs were distributed among 13 clusters (**Table 2**). Four clusters (Cluster 1, 3, 5, and 9) contained at least two OMDEGs and accounted for more than half of OMDEGs. The expression pattern of cluster 1 revealed a sustained downward trend after spawning, while the expression pattern of cluster 3 showed a flattening trend before 18h and a sharp decline after 18h (**Fig 3**). The expression trend of genes in cluster 5 continuously increased from 0 h to 24 h (**Fig 3**). The expression trend of genes in cluster 9 increased from 12 h to 18 h and then decreased after 18h (**Fig 3**). All clusters including DEGs are listed in **S4 Table**.

**Table 2:** The distribution of OMDEGs among the clusters.

Cluster	No. of OMDEGs	No. of total DEGs	Orthologous maternal DEGs
**1**	4	294	BGIBMGA010673, BGIBMGA006904, BGIBMGA005370, BGIBMGA001950
**3**	6	507	BGIBMGA013421, BGIBMGA005594, BGIBMGA003747, BGIBMGA002655, BGIBMGA008249, BGIBMGA014089
**5**	2	276	BGIBMGA004822, BGIBMGA010384
**6**	1	69	BGIBMGA011857
**7**	1	44	BGIBMGA012427
**8**	1	67	BGIBMGA010471
**9**	2	64	BGIBMGA012283, BGIBMGA013746
**11**	1	57	BGIBMGA003731
**12**	1	49	BGIBMGA010644
**14**	1	128	BGIBMGA003207
**15**	1	41	BGIBMGA006841
**18**	1	39	BGIBMGA003186
**23**	1	24	BGIBMGA005172

### Gene clusters of OMDEGs in the *B*. *mori* genome

The neighboring genes in the linking chromosomal locations usually have similar expression patterns and functions [23]. To investigate the genes correlated with OMDEGs, linking chromosome locations of DEGs were analyzed. The genes which were less than 50 kb from the OMDEG were defined as a gene cluster. Twenty-two gene clusters including 123 genes were identified from 18 scaffolds (**S5 Table**). Sixteen DEGs were located on 14 gene clusters and neighbored OMDEGs. Among them, BGIBMGA008251 had a close relationship with an OMDEG (BGIBMGA008249) with a similar expression pattern. The expression levels of these genes were down-regulated from 0 h to 24 h.

### GO annotation and enrichment analysis

A total of 14,623 unique genes matched proteins in the silkworm database and 8,156 (55.78%) were annotated with GO terms. The molecular functions of OMGs were filtered for further analysis. Fifty-eight OMGs were annotated into 108 GO terms about molecular functions, including 19 OMDEGs, 27 non-differential expressed OMGs and 12 unexpressed OMGs. The molecular functions of annotated OMGs are listed in **S6 Table**.

GO enrichment analysis was performed for analyzing functions of DEG expression clusters using Fisher’s exact test in Blast2GO. GO terms with *P*-value≤0.01 were considered to show significant enrichment between the DEGs (**S7 Table**).

In cluster 1, a total of 109 DEGs were enriched in 13 GO terms of molecular function (MF) and 35 DEGs were enriched in 11 GO terms of biological processes (BP). Two OMDEGs in cluster 1, including BGIBMGA010673 and BGIBMGA005370, were annotated in ATP binding, adenyl ribonucleotide binding and adenyl nucleotide binding functions. BGIBMGA005370 was involved in the protein modification process.

In cluster 3, a total of 148 DEGs were enriched in 15 GO terms of MF and 115 DEGs were enriched in 30 GO terms of BP. Three OMDEGs in cluster 3, including BGIBMGA005594, BGIBMGA008249 and BGIBMGA014089, were enriched in protein binding function and involved in the macromolecule metabolic process.

The enriched molecular functions were compared for four expression clusters (**S2 Fig)**. The results showed that genes involved in different expression clusters had distinct functions. Only cluster 1 and 3 did have four common function annotations including protein binding, ATP binding, adenyl ribonucleotide binding and adenyl nucleotide binding. Besides, cluster 5 and 9 had a common function annotation for RNA finding.

### Partly OMDEGs expressional analysis by RT-qPCR

**Fig 4:**
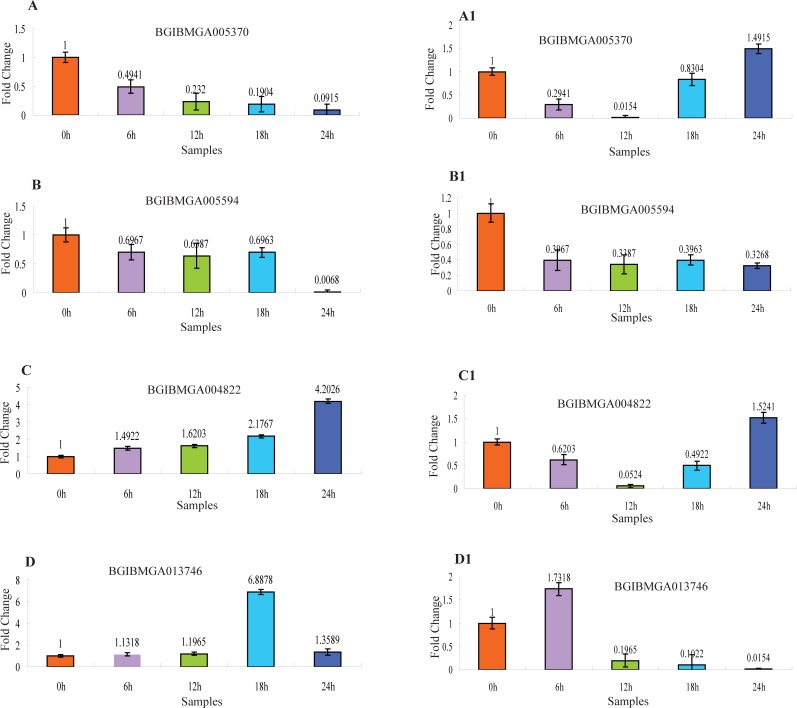
The expression analysis of four maternal genes between unfertilized and fertilized embryos using RT-qPCR. Each time point was replicated three times using independently collected samples. Error bar = 1 SD. (A), (B), (C) and (D), the samples are unfertilized embryos. (A1), (B1), (C1) and (D1), the samples are fertilized embryos. The relative BGIBMGA005370 in Cluster 1, BGIBMGA005594 in Cluster 3, BGIBMGA004822 in Cluster 5 and BGIBMGA013746 in Cluster 9 expression levels.

The gene expression levels of a part of OMDEGs was analyzed by RT-qPCR. The primers of OMDEGs were listed in **S8 Table**. One gene was selected for each expression cluster analysis. The results were shown in **Fig 4**. The expression trends of the four selected DEGs in the transcriptome were consistent with RT-qPCR results(**Fig 4A, 4B, 4C and 4D**). The expression level of BGIBMGA005370 was gradually down-regulated from 6 h to 24 h in unfertilized embryos, different from this, in fertilized embryos, the expression level of BGIBMGA005370 was sharply down-regulated from 6 h to 12 h and gradually increased from 18 h to 24 h (**Fig 4A and 4A1**). Between unfertilized and fertilized embryos, the expression trend of BGIBMGA005594 was simillar and showed significant down-regulation from 6 h to 24 h comparing to 0 h (**Fig 4B and 4B1**). The expression level of BGIBMGA004822 was gradually up-regulated from 6 h to 24 h in unfertilized embryos and down-regulated from 6 h to 18 h in fertilized embryos (**Fig 4C and 4C1**). The expression level of BGIBMGA013746 was obviously down-regulated from 12 h to 24 h in fertilized embryos (**Fig 4D1**).

## Discussion

### The functions of OMDEGs, non-differentially expressed OMGs and unexpressed OMGs

All 58 OMGs with GO annotation were divided into three groups for further analysis, including 19 OMDEGs, 27 non-differentially expressed OMGs and 12 unexpressed OMGs. The molecular function annotation showed that many OMDEGs were related to the transcriptional regulation functions, such as nucleic acid binding (BGIBMGA001950, BGIBMGA003186, BGIBMGA004822, BGIBMGA010644, BGIBMGA010673, and BGIBMGA012283), transcription factor activity (BGIBMGA013421), growth factor activity (BGIBMGA010384), methyltransferase activity (BGIBMGA011857) and so on. However, the non-differentially expressed OMGs were focused on metal ion bindings (BGIBMGA001083, BGIBMGA001789, BGIBMGA002518, BGIBMGA002519, BGIBMGA004416, and BGIBMGA003866) or some receptors (BGIBMGA000601, BGIBMGA000602, and BGIBMGA007355). In silkworm, the recombinant BmPAH (BGIBMGA003866) protein had high phenylalanine hydroxylase (PAH) activity; decreasing *BmPAH* mRNA levels in silkworm eggs reduced neonatal larval tyrosine and caused insect coloration to fail [24]. Unexpressed OMGs were similar to non-differentially expressed genes.

### Differential expressed patterns of OMGs

In this study, the expression levels of most OMGs decreased along points-in-time, such as Clusters 1 and 3. BGIBMGA003747, one of the OMDEGs, is the homologous gene of *Dmcyclin B* (the maternal gene) in *Drosophila melanogaster* and has been previously cloned from silkworm eggs [25]. The accumulation and degradation of cyclin B played a critical role in mitosis and karyokinesis [26–29]. The BmCyclin B was necessary to complete the cell cycle in BmN cells and had revealed mitotic cell divisions function in silk gland development of embryogenesis in *B*. *mori* [30, 31]. The *Bmcyclin B* mRNA, abundant in matured eggs, began to degrade sharply just after oviposition, which suggested that the penetration of sperm triggered the degradation of maternal *Bmcyclin B* mRNA in the first meiosis [25]. The expression profiling of Bmcyclin B was noticed in the middle silk glands (MSGs) during stage 24 and completely stopped after embryonic stage 25 by *in situ* hybridization and immunohistochemistry [30]. We found that the *Bmcyclin B* mRNA amount distinctly declined, which suggested the cyclin B was degraded by the ubiquitin pathway [32].

PIWI-interacting RNAs (piRNAs) play a crucial role in transposon silencing and other targets to maintain genome integrity [33, 34]. BGIBMGA011857 is homologous to TUDOR in *Drosophila*, and is known as BmTUDOR. The depletion of BmTUDOR by RNAi increased the levels of the piRNAs in *B*. *mori* [35]. The result was also in agreement with previous reports on *Drosophila* ovaries, which implicated TUDOR in piRNA biogenesis, and the TUDOR mutant contained increasing piRNA levels [36]. *Siwi* (BGIBMGA010644) had been identified as a PIWI subfamily gene in *B*. *mori;* the expression profiles of *Siwi* were abundantly expressed in the testis and ovary on day 3 of the fifth instar larvae and reached a relatively high level at 8–12 h after diapause-destined embryos oviposition [37]. In silkworm, there were two PIWI proteins, Siwi and Ago3, and two typical Heterochromatin Protein 1 (HP1) proteins, HP1a and HP1b, and silkworm PIWI proteins interact with HP1s in the nucleus acting as a chromatin regulator [38].

DNA methyltransferases (DNMTs) are some of the most important proteins associated with DNA methylation and play a major role in the silencing of gene expression, among other important functions [39]. BmDNMT-1(BGIBMGA005382) bound to unmethylated and hemimethylated DNA equally, but preferentially methylated the hemimethylated DNA, acting as a maintenance DNMT in *B*. *mori* [40]. BmDNMT-1 was highly transcribed during early embryogenesis [41]. Interestingly, knockdown of the *BmDNMT-1* gene resulted in lower hatchability [42]. In this study, *BmDNMT-1* was significantly down-regulated in the unfertilized eggs. These results indicate that *BmDNMT-1* had an important biological function during silkworm embryogenesis.

Thus, most maternal genes showed a downward trend at the beginning of development. Besides, some OMGs with an increasing trend in the first 24 h were also observed, such as OMGs in cluster 5, which indicated that these OMGs might function downstream of biological processes. When the upstream regulators finished translation, the expression levels of OMGs would be reduced over time.

Decapentaplegic (Dpp) is a subgroup of bone morphogenetic protein (BMP)-type ligands which are members of the transforming growth factor-β (TGF-β) superfamily [43]. In *Drosophila*, CHES-1-like activated TGF-β signaling by directly binding to the Dpp promoter and up-regulating Dpp expression [44]; Dpp played a significant role in immunity and several distinct stages of Drosophila development [45, 46]. BGIBMGA010384 was identified as *BmDpp*, which functioned in response to the BmNPV infection in *B*. *mori* [47]. Our data show that *BmDpp* was clearly up-regulated in unfertilized egg oviposition from 18 h to 24 h. Based on the analysis of protein domain using blastp, we found that BGIBMGA008311 was homologous to CHES-1-like in Drosophila. Interestingly, the mRNA expression level CHES-1-like was increased at 6 h of unfertilized egg oviposition in contrast to 0 h (data not shown). Therefore, CHES-1-like might act genetically upstream of Dpp and play an important role in activating TGF-β signaling in *B*. *mori*.

## Conclusion

In the current work, we detected the gene expression profile of unfertilized eggs of *B*. *mori* from five points-in-time. A total of 76 OMGs in silkworm were identified by comparison with known maternal genes of other species. More than half of the OMGs were differentially expressed and showed 13 different kinds of expression patterns. Four major expression patterns contained more than half of the OMDEGs. We also identified 22 gene clusters containing OMGs in the silkworm genome. Our findings expanded the collection of silkworm maternal genes and provided a perspective for the early embryo development of *B*. *mori*.

## Supporting information

S1 Table Tag abundances and TPM values of expressed genes. (XLSX)

S2 Table Summary of differentially expressed genes. (XLSX)

S3 Table Maternal genes of *Drosophila melanogaster* and their orthologous genes in *Bombyx mori*. (XLSX)

S4 Table Clusters of DEG expression. (XLSX)

S5 Table The gene cluster of OMDEGs. (XLSX)

S6 Table Molecular functions of orthologous maternal genes. (XLSX)

S7 Table GO enrichment results of four DEG expression clusters. (XLSX)

S8 Table Primers of genes for RT-qPCR. (XLSX)

S1 Fig All expression patterns of DEGs. The majority of expressed genes showed time-specific expression patterns and could be clustered into 27 distinct groups.
(TIFF)

S2 Fig Comparison of molecular function annotation for four expression patterns. The enriched molecular functions were compared for expression cluster 1, 3, 5 and 9. P-value was ranged from 0~1.
(TIFF)
