# Supplementary figures and images for: Identifying potential maternal genes of Bombyx mori using digital gene expression profiling
Source: PLoS One. 2018 Feb 20;13(2):e0192745. doi: 10.1371/journal.pone.0192745 (PMC5819784; doi:10.1371/journal.pone.0192745)

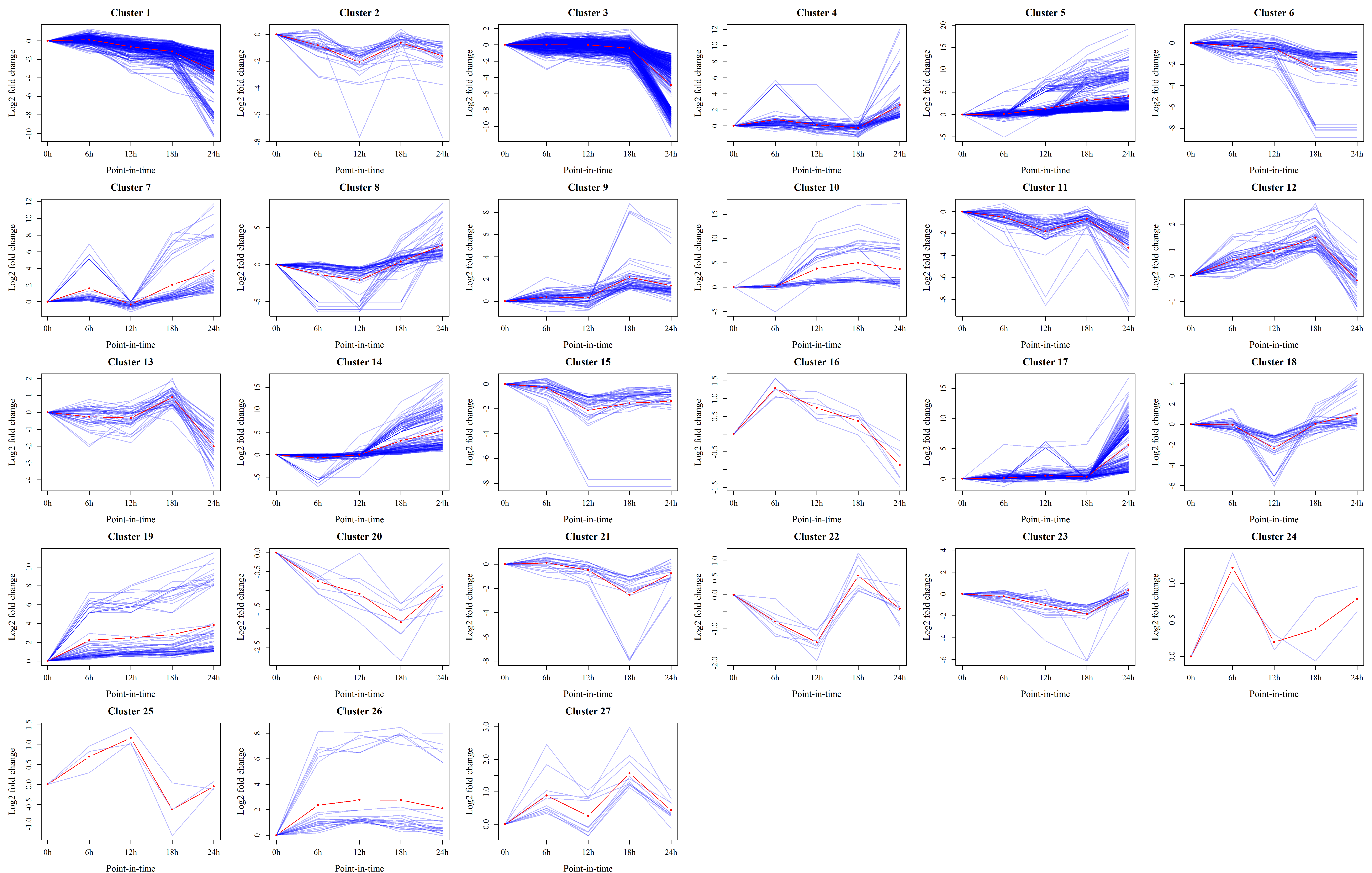

Supplement: S1 Fig — The majority of expressed genes showed time-specific expression patterns and could be clustered into 27 distinct groups. (TIFF) [file pone.0192745.s009.tiff]

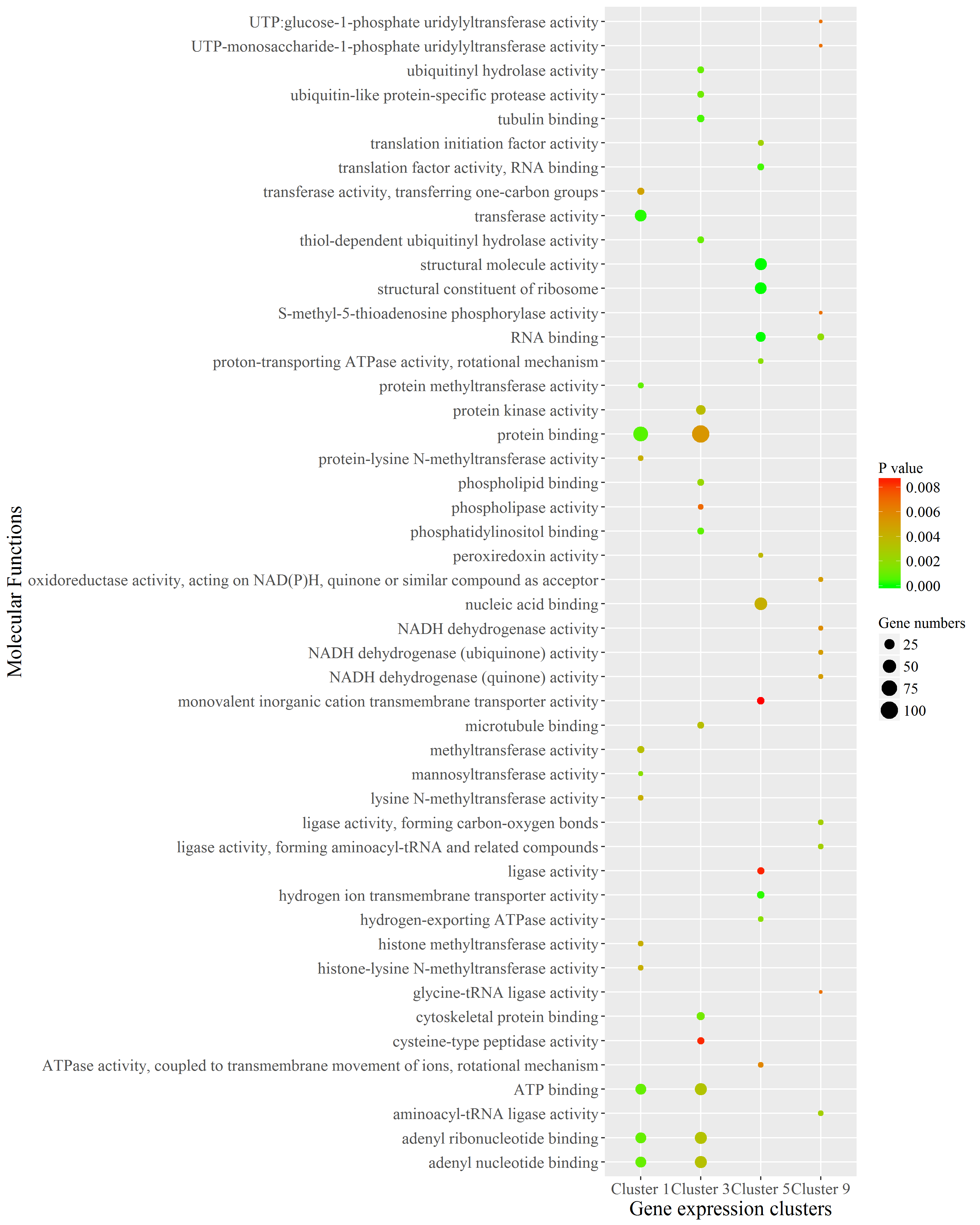

Supplement: S2 Fig — The enriched molecular functions were compared for expression cluster 1, 3, 5 and 9. P-value was ranged from 0~1. (TIFF) [file pone.0192745.s010.tiff]
